# Vitamin A and Retinoids as Mitochondrial Toxicants

**DOI:** 10.1155/2015/140267

**Published:** 2015-05-19

**Authors:** Marcos Roberto de Oliveira

**Affiliations:** Department of Chemistry, ICET, Federal University of Mato Grosso (UFMT), Avenida Fernando Correa da Costa, No. 2367, 78060-900 Cuiabá, MT, Brazil

## Abstract

Vitamin A and its derivatives, the retinoids, are micronutrient necessary for the human diet in order to maintain several cellular functions from human development to adulthood and also through aging. Furthermore, vitamin A and retinoids are utilized pharmacologically in the treatment of some diseases, as, for instance, dermatological disturbances and some types of cancer. In spite of being an essential micronutrient with clinical application, vitamin A exerts several toxic effects regarding redox environment and mitochondrial function. Moreover, decreased life quality and increased mortality rates among vitamin A supplements users have been reported. However, the exact mechanism by which vitamin A elicits its deleterious effects is not clear yet. In this review, the role of mitochondrial dysfunction in the mechanism of vitamin A-induced toxicity is discussed.

## 1. Introduction

Vitamin A (retinol) is a micronutrient present in both vegetal and animal diets [1, 2]. However, humans may be exposed to vitamin A and its derivatives (the retinoids) also pharmacologically, as in the case of therapy for dermatological disturbances, acute promyelocytic leukemia, and immunodeficiency treatment [3–9], to cite a few. During leukemia treatment, vitamin A at doses exceeding 150,000–300,000 IU/day is administrated to children at different ages and young adults [8]. Moreover, vitamin A (as retinol palmitate) is administrated to very-low-weight-preterm infants (which may born weighting 0.8–1.1 kg) at doses exceeding 8500 IU/kg·day^−1^ during weight gain therapy for undetermined period [10]. Recently, it was reported that vitamin A supplementation at 100,000 to 200,000 IU to children aged 6 to 23 months did not prevent mortality in Guinea-Bissau [11]. It is important to keep in mind that the Recommended Dietary Allowance (RDA) for vitamin A varies from 400 mcg retinol activity equivalents (RAE to conversion, please utilize 1 IU retinol = 0.3 mcg RAE) to 900 mcg RAE in males from 0 to 6 months to +51 years old and from 400 mcg RAE to 700 mcg RAE in females from the same varying age [2, 12]. Then, the levels of circulating vitamin A may be exceeded due to both inadvertent or clinical utilization.

Really, a panoply of side effects has been observed that result from vitamin A intoxication that varies from acute intoxication including, for example, headache, hepatic swelling, vomiting, and diarrhea to chronic intoxication with induction of cognitive decline in subjects at different ages, as observed in the cases of increased irritability, confusion, anxiety disorders, depression, and suicide ideation [8, 9, 13]. The exact mechanism by which vitamin A and retinoids exert such effects is not clear yet. However, it may include cell cycle disarrangement, mitochondrial dysfunction, oxidative and nitrosative stress induction, and activation of cell death signaling in different experimental models.

In this work, the effects of vitamin A and retinoids on some redox and bioenergetics parameters will be discussed focusing on mitochondrial function in different experimental models.

## 2. Vitamin A Metabolism: A Brief Overview

Vitamin A (or retinol, a diterpene) is originated from isoprene units and is characterized as an isoprenoid with a hydrocarbon chain containing a hydroxyl group at one end. The oxidation of such hydroxyl group yields retinal (an aldehyde, retinaldehyde) or retinoic acid (a carboxylic acid), the biologically active forms of retinol. In a general view, all retinoids are formed by a *β*-ionone ring and a polyunsaturated side chain and a chemical group varying from alcohol to carboxylic acid or ester as mentioned above. The presence of conjugated double bonds is noteworthy which may be in either trans-configuration or cis-configuration in the molecule of retinoids [14, 15]. Such chemical structure decreases its solubility in aqueous environments.

Vitamin A (retinol) and its derivatives, the retinoids, participate in a myriad of biological processes during animal life from development to adulthood and aging. Control of cell proliferation, differentiation, induction of cell death through apoptosis, formation and shaping of the embryo, organogenesis, and tissue homeostasis depend on physiological concentrations of vitamin A to occur adequately [9, 14–16]. Among retinoids, all-*trans* retinoic acid is better studied because it is the most biologically potent vitamin A derivative [9, 12, 14, 15]. Vitamin A and retinoids may exert their functions by binding to nuclear receptors (genomic action: induction or repression of the expression of target genes) or though regulation of signaling pathway dependent on phosphorylation of specific targets (nongenomic action: a rapid way to regulate cell events through the action of protein kinases and phosphatases) that culminate in a cellular response to such stimulus [12, 14, 15, 17–19].

Vitamin A may be obtained from both vegetal and animal diets. *β*-Carotene (an isoprenoid compound) is converted to two molecules of all-*trans*-retinal by centric oxidative cleavage and all-*trans*-retinal is reduced to all-*trans*-retinol, which may be esterified and stored in large amounts in tissues as liver, lung, and fat [12, 20, 21]. In the eyes, retinoids are converted to 11-*cis*-retinal, which is a visual chromophore that binds to opsin in order to translate light into an electrical signal [22, 23]. Esterified retinol in the form of retinol palmitate is a major source of vitamin A from diet of animal origin, as, for instance, liver, which stores the excess of vitamin A [14, 15, 20, 24].

The absorption of fat-soluble micronutrients occurs very similarly to that observed in lipids in the upper gastrointestinal tract [25, 26] after dissolution via formation of lipid droplets in both stomach and duodenum [27, 28]. The esterified forms of vitamin A (mainly retinol palmitate) are firstly hydrolyzed in the duodenum and the free form is then absorbed by the intestinal mucosa [29]. It is suggested that two pancreatic enzymes perform such hydrolysis, namely, cholesterol ester hydrolase and pancreatic lipase [30–32]. Then, the enterocyte will absorb vitamin A and carotenoids which are incorporated into micelles with other lipids from diet [25, 26]. It was reported that the efficiency of retinol absorption is around 75% [33] and 100% [34–37]. On the other hand, the efficiency of *β*-carotene absorption was estimated to be from 3% to 90% [36–38]. It was proposed that enterocytes present a specific retinol transporter that functions very efficiently [39, 40]. The absorption of carotenoids occurs mainly through passive diffusion [41].

After enterocyte uptake, retinol is esterified by lecithin retinol acyltransferase (LRAT, which utilizes phosphatidylcholine as acyl group donor) and acyl-CoA acyltransferase (ARAT), leading to the formation of retinol palmitate, retinol oleate, and retinol linoleate, among others [25, 42]. Carotenoids may follow one of these paths inside enterocytes: stay not metabolized (around 40% of provitamin A carotenoids), be cleavage generating retinal via the reaction mediated by *β*-carotene-15,15′-monooxygenase, or be cleavage by mitochondrial *β*-carotene-9′,10′-dioxygenase, which is responsible for the formation of apocarotenoids [43].

In the cytosol of the enterocyte, retinol and its derivatives (mainly retinal and retinoic acids) bind to specific proteins called cellular retinol-binding protein II (CRBP II) [25]. In other cells, as, for instance, the hepatocytes, CRBP I is responsible for free retinol transport. Additionally, the binding of retinol to CRBP is necessary to its esterification by LRAT or ARAT. In the hepatocytes, esterified retinol and retinal are also transported by CRBP I. In the plasma, retinol is transported by retinol binding protein (RBP) to general distribution to tissues [14, 15]. Retinol is converted to retinal by either microsomal or cytosolic retinol dehydrogenase (RoDH) isozymes. In turn, retinal is converted in retinoic acid by cytosolic retinal dehydrogenase (RalDH) [14, 15, 44]. Retinoic acids bind to cellular retinoic acid binding protein (CRABP) in cytosol and it is suggested that this complex migrates to nucleus to exert its effects through binding to nuclear receptors to retinoic acid (RAR or RXR) [44, 45].

Central nervous system (CNS) cells also possess nuclear receptors, CRBPs and CRABPs, as well as enzymes necessary to the local metabolism of vitamin A and derivatives. Additionally, it has been postulated that retinoids may act through a nongenomic way in different cell types, including neurons [46, 47]. The role of retinoids is not restricted to development of CNS. It has been shown that retinoids are responsible for synaptic plasticity of the hippocampus, for maintenance of dopamine signaling in mesolimbic and mesostriatal neurons, and for survival of nigrostriatal dopaminergic neurons [48–50].

## 3. The Relationship of Vitamin A and Retinoids with Biological Membranes

The hydrophobicity of vitamin A and retinoids is a chemical limiting its distribution in the aqueous compartments of the body. As mentioned above, it is necessary to bind such molecules to transport protein to increase its solubility. Indeed, vitamin A reacts with hydrophobic environments, as, for example, biological membranes, and may interfere with its physiology by perturbing phospholipid and steroids homeostasis. It was previously demonstrated that retinol induced hemolysis by penetrating rabbit erythrocytes and disrupting physical structure of the membranes [51]. According to the authors, such effect did not depend on oxidation of retinol and formation of free radicals. However, it was observed that cotreatment with vitamin E alleviated hemolysis. In that work, it was suggested that vitamin E did act by decreasing permeability and fluidity and not through its antioxidant capacity. In other works, the authors found that the retinol-induced hemolysis was dependent on hydroxyl radical formation [52]. Goodall et al. demonstrated that retinol and retinoids (retinaldehyde, *α*-retinoic acid, iso-13-retinol, and retinyl acetate) induced cell fusion, hemolysis, and swelling of mitochondria [53]. Actually, intravascular administration of all-*trans* retinoic acid to patients under treatment of acute promyelocytic leukaemia induced hemolysis and complicated the continuation of this clinical procedure [54].

Overall, such findings indicate a potential ability of vitamin A and its derivatives to negatively interact with biological membranes, an event that may lead to organelle stress, as, for instance, mitochondrial dysfunction, and to cell apoptosis or necrosis.

## 4. The Effects of Vitamin A and Its Derivatives on Mitochondrial Membranes and Organelle Physiology

### 4.1. *In Vitro* Effects of Vitamin A on Mitochondria

As previously mentioned, retinol induced mitochondrial swelling and disrupted membrane organization in* in vitro* assays [51–54]. Rigobello et al. [55] did demonstrate that different retinoic acids (namely, all-*trans*, 9-*cis*, and 13-*cis* retinoic acids) were able to induce swelling of the organelle isolated from rat liver. All the retinoids tested induced membrane permeability transition (which was observed as swelling) and decreased membrane potential. Interestingly, neither EGTA (Ca^2+^ ion chelating agent) nor cyclosporin A (CsA) inhibited the effects elicited by 13-*cis* retinoic acid. Additionally, 13-*cis* retinoic acid induced cytochrome c release from the organelle, an event that is necessary to trigger the intrinsic apoptotic pathway [56]. Later, it was reported that retinol also altered mitochondrial structure by inducing swelling and lipid peroxidation in mitochondrial membranes* in vitro* [57]. In addition, retinol induced cytochrome c release and increased superoxide anion radical (O_2_^−•^) production in a dose-dependent pattern. When analyzed together, such results indicate part of the mechanism by which retinol and retinoids may trigger cell death through the mitochondrial/intrinsic pathway. Also, it demonstrates that vitamin A may exert prooxidant effects by altering mitochondrial function and favoring electron leakage from mitochondria, leading to increased free radical generation.

Really, it was demonstrated that retinol induced apoptosis in cultured Sertoli cells by a mitochondria-dependent pathway [58]. In such work, the researchers found that retinol induced a decrease in cell viability and ATP content and increased O_2_^−•^ formation. Additionally, increased cytochrome c release to the cytosol and, consequently, increased caspase-3/7 enzyme activity were observed. Then, from isolated mitochondria assays to cultured cells, the deleterious effects of vitamin A on mitochondria may be observed. Such negative action of this vitamin and its derivatives on mitochondrial function and/or dynamics may result in cell death. The release of cytochrome c to cytosol may lead to two important processes: increased O_2_^−•^ production and apoptosis through formation of the apoptosome. However, apoptosis is dependent on sufficient ATP levels because the apoptosome consumes ATP (or dATP) to cleave and activate caspases. Then, deregulated cytochrome c release may lead the cells to die by necrosis, which induces inflammation, an even more deleterious process to cell viability (for a review, please see [56]).

Silva et al. reported that acitretin (a synthetic retinoid that is used in the treatment of severe extensive psoriasis) at 5–20 *μ*M altered the function of rat liver mitochondria* in vitro* [59]. The authors found impaired phosphorylation capacity, decreased ATP levels and adenine nucleotide translocase (ANT) content, and Ca^2+^-induced mPTP (mitochondrial permeability transition pore). On the other hand, decreased membrane potential was not observed. Surprisingly, such effects were not reverted by the cotreatment with thiol group reductants or other antioxidant agents, showing, at least in part, that a redox mechanism did not take part in the events observed. On the other hand, mPTP was blocked by ANT ligands, as, for instance, ATP and ADP.

Recently, Sawada et al. reported that all-*trans*-retinal (10–30 *μ*M), a byproduct of the visual cycle (originated from the chromophore 11-*cis*-retinal), decreased viability of ARPE-19 cell line and induced oxidative stress-dependent Bax activation through PLC/IP3/Ca^2+^ signals and by activation of p53 following DNA damage [60]. The authors conclude that all-*trans*-retinal affected cell viability by a mechanism that increased the concentration of cytosolic Ca^2+^ ions, which lead to oxidative stress and DNA damage. In turn, it activates p53 through a mechanism dependent on phosphorylation of ser46 residue and translocation to the cytosol, where it activates Bax, triggering apoptosis. This work demonstrates the importance of maintaining the levels of all-*trans*-retinal under control in retina, since disrupted all-*trans*-retinal clearance may lead to retinopathy, as previously reported [61]. However, the authors did not investigate the role of mitochondria in the induction of apoptosis in that work.

A synthetic retinoid (ST1926) was recently tested for treating adult T-cell leukemia/lymphoma and demonstrated ability to induce growth arrest and apoptosis of T malignant cells [62]. ST1926 at 1 *μ*M for 48 hours induced apoptosis in HuT-102, MT-2, Jurkat, and Molt-4 cell lines. Even though the apoptotic mechanism depends on caspase-3, any parameter related to mitochondrial dynamics was not investigated in that work.

### 4.2. *In Vivo* Effects of Vitamin A on Mitochondria

The effects of vitamin A and retinoids on mitochondrial function were well investigated* in vitro*. However, recently it was demonstrated that intragastric (gavage) vitamin A supplementation at pharmacological doses (from 1,000 to 9,000 IU/kg·day^−1^) for 3, 7, or 28 days induced redox (Table 1) and bioenergetics (Table 2) impairments in rat brain regions and other tissues of adult male Wistar rats, as discussed below. Additionally, some abnormalities in behavioral tasks were observed, as, for example, in the open field and light-dark box [63–66].

**Figure 1:**
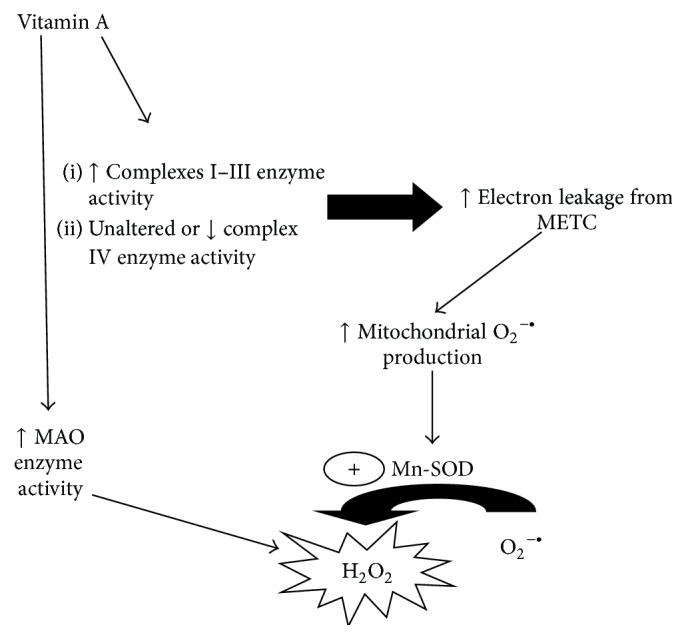
A schematic diagram summarizing the effects of* in vivo* vitamin A supplementation on mitochondrial function regarding the mitochondrial electron transfer chain (METC) enzyme activity. Mitochondrial dysfunction may lead to increased O_2_^−•^ production through electron leakage and partial reduction of O_2_. Mn-SOD converts O_2_^−•^ to H_2_O_2_ and, together with MAO, favors an increase in the levels of H_2_O_2_ in different cell types (please see text for details). H_2_O_2_ is able to react with iron ions generating ^•^OH (the most powerful ROS) through Fenton chemistry reaction (not shown), for example, leading to widespread redox disturbances.

Vitamin A supplementation increased mitochondrial superoxide anion radical (O_2_^−•^) production (Table 3) and induced lipid peroxidation, protein carbonylation and nitration, and oxidation of protein thiol groups in mitochondrial membranes isolated from rat cerebral cortex, cerebellum, substantia nigra, striatum, frontal cortex, and hypothalamus [67–69, 76, 78]. In the same rat brain areas, increased complex I–III enzyme activity was observed [67–69, 76, 78]. However, a proportional increase in the following complexes of the mitochondrial electron transfer chain (METC) was not found as expected. For example, vitamin A supplementation induced a decrease in complex IV enzyme activity in rat cerebellum, striatum, and hypothalamus [67, 68, 76]. On the other hand, any change in some complexes activities was not observed as follows: complexes II, II-III and succinate dehydrogenase (SDH) (cerebellum) [76]; complex IV (substantia nigra) [67]; complexes II-III and SDH (striatum, hypothalamus) ([67], [68], resp.); complexes II-III, SDH, and complex IV (frontal cortex) [69] (Table 2). Such impairment in electron flux between mitochondrial complexes may favor electron leakage from the electron transfer chain, since the electron flux is higher between some complexes, but the reduction of O_2_ to water is not occurring at the same rate due to unaltered or even decreased complex IV enzyme activity (Figure 1). Also, more O_2_ is available to react with electron donors and becomes O_2_^−•^ [79, 80]. Furthermore, increased complexes I–III, II-III, and II and SDH and complex IV enzyme activities were also reported in the liver of the animals that receive vitamin A supplementation at clinical doses for 28 days [72]. These findings are different from that observed in brain regions of the animals that received vitamin A for the same period, as described above, since it was demonstrated that complex IV enzyme activity was increased at a very similar rate when compared to complexes I–III in rat liver. However, such increment in the electron flux between the electron transfer chain (ETC) complexes was accompanied by a proportional increase in O_2_^−•^ production (Table 3). This result may suggest that O_2_^−•^ is being produced by mitochondria isolated from vitamin A-treated rats by a mechanism that is not obligatorily associated with uncoupling of the ETC activity.

More recently, it was published that vitamin A supplementation induced an increase in total 3-nitrotyrosine content in rat cerebral cortex, hippocampus,* substantia nigra*,* striatum*, hypothalamus, heart, and lung [67, 68, 71, 74, 75, 81]. In addition, increased 3-nitrotyrosine content in proteins located in the mitochondrial membranes isolated from frontal cortex, hippocampus, heart, and lung of vitamin A-treated rats was reported [69, 71, 74, 75] (Table 1). The formation of 3-nitrotyrosine is a consequence of increased levels of O_2_^−•^ and NO^•^, which give rise to peroxynitrite (ONOO^−^), that may react with tyrosine residues in proteins leading to the formation of 3-nitrotyrosine. Additionally, ONOO^−^ may give rise to peroxynitrous acid (ONOOH), which produces nitryl cation (NO^2+^), nitrogen dioxide radical (^•^NO_2_), and hydroxyl radical (^•^OH) through homolytic fission reaction [82, 83]. At least in part, the increase in 3-nitrotyrosine content may be explained by the increase in mitochondrial O_2_^−•^ production elicited directly or indirectly by vitamin A supplementation. In order to investigate whether NO^•^ production (as indirectly assessed through 3-nitrotyrosine formation) participates in mitochondrial dysfunction and behavioral disturbances observed in the experimental model of vitamin A supplementation, the role of a cotreatment with L-NG-nitroarginine methyl ester was tested (L-NAME; 30 mg/kg four times a week), a nonspecific nitric oxide synthase (NOS) inhibitor, on such parameters. Interestingly, L-NAME cotreatment did not exert any effect on the redox unbalance elicited by vitamin A on rat frontal cortex, hippocampus, substantia nigra, and striatum [77].

It was previously described that increased formation rates of 3-nitrotyrosine favor protein aggregation, which may lead to serious consequences regarding mitochondrial function, such as import of molecules (from metabolic substrates to proteins necessary to the ETC function, among others) from cytosol and other complex processes as mitochondrial fusion and fission. Both *α*-synuclein and *α*-tubulin may be nitrated and form protein aggregates that accumulate in cytoplasm [84–86]. *α*-Synuclein has been implicated in the mechanism behind the pathogenesis of neurodegenerative synucleinopathies [84, 87]. Recently, it was shown that *α*-synuclein may interact negatively with mitochondria causing it to lose transmembrane potential and decrease phosphorylation capacity [88]. In fact, *α*-synuclein may bind to the inner mitochondrial membrane in *α*-helical conformation [89]. Interestingly, increased levels of *α*-synuclein, but unaltered levels of *β*-synuclein, in brain regions of vitamin A-treated rats were demonstrated [67, 71, 77]. However, neither alterations in *α*-synuclein structure nor interactions of such protein with mitochondria in the experimental model of vitamin A supplementation were investigated.

**Figure 2:**
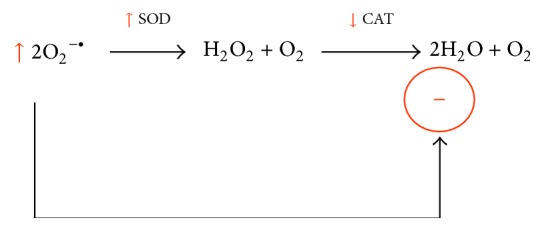
Unbalanced SOD/CAT ratio resulting in increased H_2_O_2_ production. Additionally, increased O_2_^−•^ levels inhibit CAT enzyme activity allosterically leading to even more high H_2_O_2_ concentration due to accumulation of this ROS.

**Figure 3:**
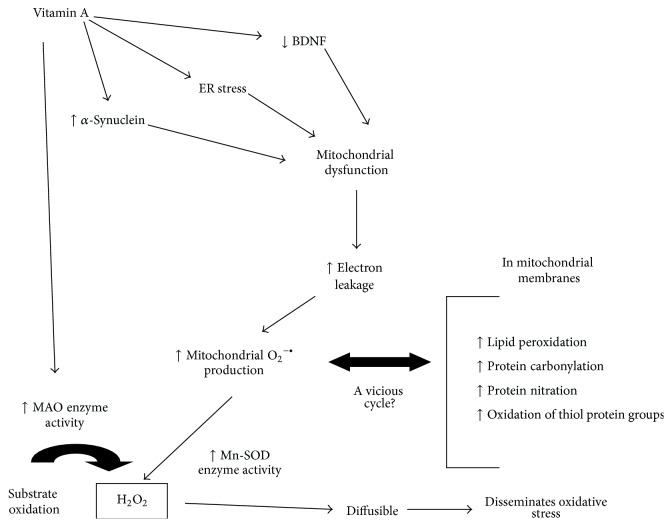
A general view of the effects of* in vivo* vitamin A supplementation in an animal experimental model. It has been hypothesized that vitamin A may induce mitochondrial dysfunction by different ways as follows: (1) by decreasing BDNF levels, (2) by inducing ER stress and calcium ion metabolism deregulation, and/or (3) by increasing *α*-synuclein levels. The increased O_2_^−•^ levels may induce redox unbalance in the organelle that, in turn, may generate more O_2_^−•^ in a vicious cycle. Increased H_2_O_2_ production (by Mn-SOD and MAO enzymes) may disseminate redox impairment from one region to another.

On the other hand, it was shown that vitamin A supplementation for 28 days increased monoamine oxidase (MAO) enzyme activity in both areas of the nigrostriatal axis and hippocampus [71, 77, 90] (Table 3). MAO is responsible for the chemical inactivation of dopamine and serotonin and produces H_2_O_2_ in such reaction [87, 91]. MAO is located in the outer mitochondrial membrane facing the cytosol, but H_2_O_2_ is a membrane soluble ROS and may enter mitochondria or other organelles [91]. Taken together, such data indicate mitochondria as an important source of H_2_O_2_, since manganese-superoxide dismutase (Mn-SOD; mitochondrial) and MAO enzyme activities were found increased in the hippocampus and nigrostriatal axis of vitamin A-treated rats [71, 90] (Table 3). H_2_O_2_, which is also water soluble, may diffuse to places far away from its origin, disseminating the redox impairment from one cellular environment to another [92–97] (Figure 1). Interestingly, CAT enzyme activity was found either unaltered or decreased in brain areas of vitamin A-exposed rats [63, 64, 66]. Such finding suggests that an impairment exists also on the ratio between SOD and CAT enzyme activities, which may favor an increase in H_2_O_2_ production. Furthermore, accumulated O_2_^−•^ is able to inhibit CAT enzyme activity, as well as other enzymes [98] (Figure 2). Then, it may be suggested that, in the experimental model of vitamin A supplementation, mitochondria is a biological source of H_2_O_2_ in some rat brain regions and such effect may be linked to the oxidative stress observed in some reports (Figure 3).

In addition to a possible H_2_O_2_ generation increase, increased glutathione S-transferase (GST; an enzyme that is responsible for phase II detoxification reactions of conjugation in several cell types) enzyme activity in the vitamin A supplementation experimental model was detected [67, 76]. Such enzyme consumes reduced glutathione (GSH) to produce more polar xenobiotics that are easily excreted from cells [99]. By consuming GSH at increased rates, it may facilitate the perpetuation of H_2_O_2_ prooxidant signal, since GSH is utilized by GPx in the conversion of H_2_O_2_ to water [87, 100, 101]. In the nigrostriatal axis, there is a high Fe^2+^ content that may react with H_2_O_2_ through Fenton chemistry reaction in cases of hypervitaminosis A, for example, leading to increased production of ^•^OH, the most powerful free radical in biological systems [87, 102, 103]. Indeed, it may facilitate dopaminergic neuronal death by either apoptosis or necrosis, leading detrimental effects on movement control, as observed in patients suffering from Parkinson's disease [104, 105]. Although redox impairment was found in such rat brain areas, any alteration regarding cellular markers of cell death was not observed, such as caspase-3 or caspase-8 enzyme activity [67–69, 78, 90].

### 4.3. *Ex Vivo* Effects of Vitamin A on Mitochondria

Vitamin A supplementation at clinical doses for 3 or 7 days induced several prooxidant effects also on rat liver, which is the main site of vitamin A storage in mammals [14, 15, 47]. It was observed that vitamin A supplementation (1,000 to 9,000 IU/kg·day^−1^) for 3 or 7 days induced oxidative stress in mitochondrial membranes and increased O_2_^−•^ production [73]. Also, increased complexes I–III enzyme activity was demonstrated without any effect on complexes II-III and IV. However, the more surprising in that work is the fact that intact mitochondria isolated from the liver of the animals that received vitamin A supplementation were found to be more sensitive to an incubation of 10 minutes with CaCl_2_ at low concentration (75 *μ*M;* ex vivo* assay). Calcium ions mediate mitochondrial dysfunction by increasing reactive oxygen species (ROS) production and triggering mPTP, resulting in apoptosis as reviewed elsewhere [106–108]. A 2.5- to 2.9-fold increase in lipid peroxidation levels in the mitochondria isolated from vitamin A-treated rats when exposed to CaCl_2_ was detected. Similar effects were seen when protein carbonylation and thiol oxidation markers were quantified in such experimental model. Cotreatment with DTT, GSH, superoxide dismutase (SOD), or catalase (CAT) did decrease the prooxidant effect induced by CaCl_2_. Neither CsA nor bongkrekic acid (BKA) (mPTP inhibitors) did alter the effect induced by CaCl_2_ [73]. Then, such data suggest that the prooxidant effects that appeared after exposure to CaCl_2_ are not related to mPTP formation. Additionally, CaCl_2_ amplified O_2_^−•^ production in intact mitochondria isolated from vitamin A-treated animals. However, only cotreatment with GSH or SOD did decrease CaCl_2_-induced O_2_^−•^ production [73]. Then, it may be concluded that* in vivo* vitamin A supplementation increased the* ex vivo* mitochondrial susceptibility to a challenge that indirectly induces a prooxidant state in the organelle. However, it was not associated with mPTP formation, as indicated through the utilization of mPTP inhibitors. At least in part, some of the findings presented above are similar to the effects elicited by the treatment with a synthetic retinoid (acitretin) on mitochondrial function* in vitro* [59].

**Figure 4:**
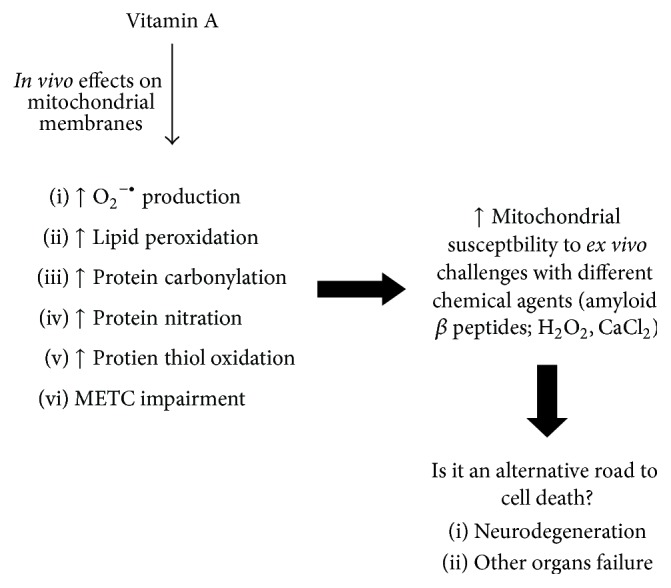
A general view of the consequences of* in vivo* vitamin A supplementation on the susceptibility of mitochondria to* ex vivo* challenges with different chemical agents. Mitochondria isolated from vitamin A-treated rats are more sensitive to different chemical insults including amyloid *β*, H_2_O_2_, and CaCl_2_, as discussed in the text.

The effects of vitamin A supplementation on a mitochondrial challenging with CaCl_2_ in the case of rat liver analyses were discussed above. However, it was also investigated whether* in vivo* vitamin A supplementation altered brain mitochondria response to an* ex vivo* challenge with H_2_O_2_ or *β*-amyloid peptide_1–40_ and peptide_1–42_ [70, 90]. As expected, vitamin A supplementation increased the susceptibility of mitochondria (isolated from the nigrostriatal axis and from frontal cortex and hippocampus) to H_2_O_2_ (a ROS) and to *β*-amyloid peptide_1–40_ and peptide_1–42_ (which accumulate at both extra- and intraneuronal environments in the case of Alzheimer's disease) [87]. *β*-Amyloid peptide_1–40_ and peptide_1–42_, which may accumulate in the extracellular environment, also are able to enter neurons and interact with organelles, such as mitochondria, leading to membrane rupture, among other effects, and general dysfunction [109–113]. It is an important finding demonstrating that even recommended doses of vitamin A (which have been considered to be secure to humans) facilitate mitochondrial damage when such organelles are exposed to reactive molecules (with or without radical nature) (Figure 4).

### 4.4. Other Evidences of Vitamin A-Induced Toxicity on Mammalian Mitochondria

It was also observed that vitamin A supplementation (1,000–9,000 IU/kg·day^−1^ for 28 days) induced a decrease in the levels of brain-derived neurotrophic factor (BDNF) in rat hippocampus [71]. BDNF is a major neurotrophin in the mammalian brain and is involved in the induction of neuronal proliferation and maintenance of neuron survival [114–116]. Furthermore, BDNF may signal mitochondrial biogenesis in different cell types including neurons [117, 118]. Then, BDNF is also responsible, at least in part, for maintaining ATP homeostasis in mammalian cells. However, a causal link between mitochondrial dysfunction and deregulated BDNF levels was not established yet.

Some evidences point to vitamin A as an inducer of endoplasmic reticulum (ER) stress since increased BiP/Grp78 levels in the hippocampus of vitamin A-treated rats was reported [71]. BiP (a protein chaperone) is a major regulator of ER function and participates, for example, in protein folding and assembly, binding to Ca^2+^ ions, and controlling ER stress sensors activation [130, 131]. Whether vitamin A or one of its derivatives alter ER function was not demonstrated yet; but by inducing ER stress, vitamin A may deregulate Ca^2+^ ions homeostasis, which may lead to mitochondrial dysfunction and cell death [132] (Figure 3).

## 5. Clinical Hypothesis of the Impact of Hypervitaminosis A on Human Health

Mitochondrial dysfunction gives rise to a myriad of consequences. It includes bioenergetics deficits, increased production of reactive oxygen or nitrogen species (ROS and RNS, resp.), and apoptosis or necrosis. Then, it is very important to maintain mitochondrial homeostasis to avoid loss of cellular quality and death by mechanisms that may culminate in inflammation, for example.

It has been shown that retinoids possess an ability to alter cell cycle and to induce apoptosis in some experimental models. It was published that the treatment of adult mice with 13-cis-retinoic acid at 1 mg/kg·day^−1^ (a clinical dose commonly applied in the treatment of nodular acne) for 1–6 weeks suppressed hippocampal cell division (neurogenesis) and, consequently, decreased capacity to learn in behavioral task [133]. Accordingly, Sakai et al. demonstrated increased cell loss in the hippocampus of mice treated for 3 weeks with 13-cis-retinoic acid at 1 mg/kg·day^−1^ [134]. The mechanism by which 13-*cis*-retinoic acid altered neurogenesis and induced cell death in mice hippocampus is not clear, but it has been reported that this retinoid may trigger apoptosis through activation of caspase-3 and by modulating bcl2 and p53 gene expression in melanoma cells [135]. Reinforcing the finding that a retinoid may induce negative consequences to hippocampal function, it was reported that vitamin A supplementation with retinol palmitate induced anxiety-like behavior in adult rats [63]. Anxiety is a behavior closely related to alterations in the function of hippocampus and significantly decreases human life quality [136–138]. Furthermore, studies in humans demonstrated that the use of 13-*cis*-retinoic acid (as treatment to nodular acne) decreased metabolism in orbitofrontal cortex, a region associated with depression [119]. Indeed, there is a strong body of evidence showing that 13-*cis*-retinoic acid (isotretinoin) induced depression and increased both suicide ideation and suicide rates among some patients under such treatment [120–124]. However, it remains to be elucidated whether there is a causal link between bioenergetics impairment and neuronal dysfunction that leads to detrimental alteration in human behavior.

In fact, the capacity of retinoids to induce mitochondrial dysfunction and cell death has been utilized pharmacologically as a strategy to treat several human diseases from dermatological disturbances to some types of cancer (Table 4). On the other hand, it is not clear whether a vitamin A overload would be beneficial to cells under constant stress and low antioxidant defenses, as, for instance, neurons [87, 139, 140]. Increased cell death rates are observed in the case of Parkinson's disease and Alzheimer's disease [87], and increased ingestion or other forms of exposure to such vitamin may favor a more drastic situation with accelerated neuronal loss and increased neuroinflammation. Really, it has been reported that vitamin supplements utilization (including vitamin A and carotenoids) by well-nourished subjects may increase risk of mortality among them [125–127]. Indeed, the ingestion of antioxidant supplements in the primary prevention of chronic diseases or mortality in agreement with recent dietary guidelines is not suggested [127]. Additionally, it is alarming that the combination of *β*-carotene (30 mg; vitamin A precursor from vegetal diet) and retinol palmitate (25,000 IU) supplementation increased lung cancer incidence among men and women in a clinical trial that has to be stopped due to increased lung cancer and death among the volunteers [141]. However, the mechanisms by which vitamin A and retinoids, among other lipophilic vitamins, alter cell function leading to death remain to be elucidated.

## 6. Conclusion

Vitamin A and its derivatives, the retinoids, disrupt mitochondrial function by a mechanism that is not completely understood. However, it accounts with impaired electron flux between the complexes of the METC, increased ROS production, and induction of oxidative and nitrosative stress to mitochondrial membranes. Additionally, vitamin A and retinoids alter the mitochondrial structure by causing swelling of the organelle. More investigations are needed to elucidate how vitamin A and retinoids affect mitochondria and whether there is a causative link between such event and the clinical manifestations observed both experimentally and in humans.

Then, even though more investigations in this field are necessary, it is more secure to take some caution when vitamin A has been ingested at higher than recommended levels by individuals with familial history of neurodegenerative diseases, for instance, Alzheimer's disease and Parkinson's disease, or are already affected by such irreversible disorders. Really, the fact that vitamin A increased susceptibility of mitochondria to some common cellular stress inducer agents (CaCl_2_ and H_2_O_2_ and not only *β*-amyloid peptide_1–40_ and peptide_1–42_) must be considered in the case of utilization of such micronutrient as supplement or fortified food in any case of disease, not only those from neuronal origin.

Overall, caution must be taken when utilizing vitamin A or its derivatives in some specific conditions, since such molecules regulate cell cycle and cell fate (survival or death) by different ways and its toxic effects may also lead to irreversible damage.

## Figures and Tables

**Table 1:** Summary of the *in vivo* effects of subacute vitamin A supplementation on mitochondrial membranes parameters.

Sample	Lipid peroxidation	Protein carbonylation	Protein nitration	Protein thiol content	Reference
Cerebral cortex	↑	↑	Not measured	↓	[65]
Cerebellum	↑	↑	Not measured	↓	[65]
Substantia nigra	↑	Not measured	Not measured	Not measured	[67]
Striatum	↑	Not measured	Not measured	Not measured	[67]
Hypothalamus	↑	Not measured	Not measured	Not measured	[68]
Frontal cortex	↑	↑	↑	Unaltered	[69, 70]
Hippocampus	↑	↑	↑	Unaltered	[70, 71]
Liver	↑	↑	Not measured	Unaltered	[72, 73]
Heart	Not measured	Not measured	↑	Not measured	[74]
Lung	↑	↑	↑	Unaltered	[75]

Adult male rats were treated with vitamin A supplementation (1,000–9,000 IU/kg/day) subacutely (see text for details).

**Table 2:** Summary of *in vivo* effects of subacute vitamin A supplementation on mitochondrial function parameters.

Sample	Complexes I–III	Complexes II-III	Complexes II + SDH	Complex IV	Reference
Cerebral cortex	Not measured	Not measured	Not measured	Not measured	—
Cerebellum	↑	Unaltered	Unaltered	↓	[76]
Substantia nigra	↑	↑	↑	Unaltered	[67]
Striatum	↑	Unaltered	Unaltered	↓	[67]
Hypothalamus	↑	Unaltered	Unaltered	↓	[68]
Frontal cortex	↑	Unaltered	Unaltered	Unaltered	[69, 70]
Hippocampus	↑	Unaltered	Unaltered	↓	[70, 71]
Liver	↑	↑	↑	↑	[72, 73]
Heart	↓	↓	↓	Not measured	[74]
Lung	↑	↑	↑	Not measured	[75]

Adult male rats were treated with vitamin A supplementation (1,000–9,000 IU/kg/day) subacutely (see text for details).

**Table 3:** Summary of *in vivo* effects of subacute vitamin A supplementation on mitochondrial redox parameters.

Sample	Superoxide anion radical	Mn-SOD enzyme activity	MAO enzyme activity	Reference
Cerebral cortex	↑	Not measured	Not measured	[65]
Cerebellum	↑	Not measured	Not measured	[65]
Substantia nigra	↑	↑	Unaltered	[67]
Striatum	↑	↑	↑	[67]
Hypothalamus	↑	Not measured	Not measured	[68]
Frontal cortex	↑	↑	↑	[77]
Hippocampus	↑	↑	↑	[77]
Liver	↑	Not measured	Not measured	[73]
Heart	Not measured	Not measured	Not measured	—
Lung	↑	Not measured	Not measured	[75]

Adult male rats were treated with vitamin A supplementation subacutely (see text for details).

**Table 4:** Clinical utilization of vitamin A and retinoids.

Retinoid	Utilization	Reference
Various	Prevention of infectious diseases	[4]
Retinol palmitate	Treatment of acute promyelocytic leukemia	[5, 7]
Retinol palmitate	Treatment of acute nonlymphocytic leukemia	[6]
Various	Weight gain therapy in preterm infants	[10]
Retinol palmitate/acetate	Immunotherapy (with vaccination)	[11]
Isotretinoin	Acne therapy	[119–124]
Various	Antioxidant therapy, increased longevity (supplements)	[125–127]
Retinyl esters	Treatment of infants born from HIV-positive women (immunodeficiency therapy)	[128]
Various	Antioxidant therapy in heart disease	[16]
Various	Utilization in general dermatology	[129]

## Abbreviations

ANT: Adenine nucleotide translocase
ARAT: Acyl-CoA acyltransferase
BDNF: Brain-derived neurotrophic factor
BKA: Bongkrekic acid
CNS: Central nervous system
CRABP: Cellular retinoic acid binding protein
CRBP I: Cellular retinol-binding protein I
CRBP II: Cellular retinol-binding protein II
CAT: Catalase
CsA: Cyclosporin A
ER: Endoplasmic reticulum
ETC: Electron transfer chain
GSH: Glutathione
GST: Glutathione S-transferase
L-NAME: L-NG-nitroarginine methyl ester
LRAT: Lecithin retinol acyltransferase
MAO: Monoamine oxidase
METC: Mitochondrial electron transfer chain
Mn-SOD: Manganese-superoxide dismutase
mPTP: Mitochondrial permeability transition pore
NOS: Nitric oxide synthase
RAE: Retinol activity equivalents
RalDH: Retinal dehydrogenase
RAR: Retinoic acid receptor
RBP: Retinol binding protein
RDA: Recommended Dietary Allowance
RNS: Reactive nitrogen species
RoDH: Retinol dehydrogenase
ROS: Reactive oxygen species
SDH: Succinate dehydrogenase
SOD: Superoxide dismutase.
